# Fecal Microbiota Transplantation: A Potential Tool for Treatment of Human Female Reproductive Tract Diseases

**DOI:** 10.3389/fimmu.2019.02653

**Published:** 2019-11-26

**Authors:** Gianluca Quaranta, Maurizio Sanguinetti, Luca Masucci

**Affiliations:** ^1^Istituto di Microbiologia, Università Cattolica del Sacro Cuore, Rome, Italy; ^2^Dipartimento Scienze di Laboratorio e Infettivologiche, Fondazione Policlinico Universitario A. Gemelli IRCCS, Rome, Italy

**Keywords:** cervical-vaginal microbiota, uterine microbiota, gut micobiota, fecal microbiota transplant (FMT), probiotics, immunmodulation, next generation sequencing (NGS)

## Abstract

The gastro-intestinal tract is an extensive organ involved in several activities, with a crucial role in immunity. Billions of commensal and transient microorganisms, known as the gut microbiota, and potential pathogens, which are constantly stimulating intestinal immunity, colonize the intestinal epithelial surface. The gut microbiota may be regarded as analogous to a solid organ with multiple different functions. In the last decade, many studies have demonstrated that intestinal bacteria can be a decisive factor in the health-disease balance of the intestine, and they can also be responsible for illnesses in other locations. For this reason, fecal microbiota transplantation (FMT) represents an important therapeutic option for *Clostridium difficile* infections and hold promise for different clinical conditions, such as multiple sclerosis, autism, obesity, and other systemic diseases. FMT consists of the infusion of a fecal suspension from a healthy donor to a recipient in order to restore gut flora alterations. Similar to the gut, the female reproductive tract is an example of a very complex biological ecosystem. Recent studies indicate a possible relationship between the gut and female tract microbiota, associating specific intestinal bacteria patterns with genital female diseases, such as polycystic ovary syndrome (PCOS), endometriosis and bacterial vaginosis (BV). FMT could represent a potential innovative treatment option in this field.

## Introduction

The gut microbiota, which is composed of 10^13^-10^14^ bacterial cells (1), has multiple different functions. In the last decade, studies have shown that intestinal bacteria can interact with other organs and be a decisive factor in health/disease balance beyond just the intestine (2). The gut and vaginal microbiota are examples of very complex biological ecosystems. The female reproductive tract has developed unique structures, such as the vagina and uterus. While the vagina hosts trillions of bacteria, the upper reproductive tract remains largely unexplored and has generally been considered sterile. The vaginal microbiota interacts with the immune system. Despite being one of the simplest commensal bacterial communities in the human body, we are only beginning to appreciate its complex dynamic nature and modulation of host immunity (3). Toll-like receptor (TLR)-mediated signaling, for example, regulates mucin secretions, contributing to local bacteria colonization (4). The relationship between the gut and female reproductive tract microbiota has been studied in-depth (5). Driving microbial colonization in the gut could be a very interesting approach for the treatment of many diseases. Fecal microbiota transplantation (FMT) is an important therapeutic option for *Clostridium difficile* [or *Clostridioides difficile*-like, as reported by Oren et al. (6)] infections (CDI) (7). Promising findings suggest that FMT may also play a role in the management of genital female disorders associated with microbiota alterations.

## Human Gut Microbiota

Human microbiota is composed of archea, bacteria, yeasts, fungi, viruses, and protists (8), whose composition has not been completely described also because there is a substantial inter-individual. Human gut microbiota is a complex ecosystem with several functions integrated into the host organism, interacting with metabolic, immune, and nutrient absorption activities. The gastrointestinal tract harbors ~10^13^-10^14^ bacterial cells (1, 9), consisting of strict anaerobes that outnumber the facultative anaerobes and aerobes by a factor of two to three; these include Firmicutes, Bacteroidetes, and Proteobacteria, while Actinobacteria contribute less to the total bacterial composition. Bacteroidetes include the genera *Prevotella* and *Bacteroides*; the phylum Actinobacteria includes *Bifidobacterium*; and the phylum Firmicutes includes *Clostridium* clusters and members of *Eubacterium, Faecalibacterium, Roseburia*, and *Ruminococcus* (10). The presence of many different bacterial species is crucial for defining the role of gut microbiota in various metabolic pathways. Gut microbiota is a dynamic system that changes and evolves during our lifetime according to anatomical, dietary, environmental, pathological, and pharmacological factors (e.g., the use of antibiotics, probiotics) (11). This variability in terms of bacterial species is distributed throughout the various districts of the gastrointestinal system. Starting from the upper gastro-intestinal tract, the throat and distal esophagus, the predominant genera are *Streptococcus, Prevotella, Actinomyces, Gemella, Rothia, Granulicatella, Haemophilus*, and *Veillonella* (12). In the stomach, microbial diversity depends upon the presence and absence of *Helicobacter pylori* (13, 14). A stomach lacking *H. pylori* is mainly populated by *Streptococcus* spp., *Actinomyces* spp., *Prevotella* spp., and *Gemella* spp., which are predominantly found in the throat, indicating that they may be transient residents coming from the throat (12). The recto-sigmoid colon microbiota is more complex than the jejunum, ileum, and caecum resident microbes. *Enterococci, E. coli, Klebsiella, Lactobacilli, Staphylococci*, and *Streptococci* are present in the jejunum and ileum. Most of the microbes of the jejunum and ileum are aerobes and facultative anaerobes (15). The small intestine harbors the aerobic *Enterococcus* group, *Lactobacilli, Streptococci*, and Gammaproteobacteria, while anaerobes are predominant in the large intestine. Caecal microbiota is more complex than jejunal and ileal microbiota. The caecal bacteria are predominated by *Lactobacillus, Enterococcus*, and *Escherichia coli* (16). In the recto-sigmoidal colon, strict anaerobic bacteria belonging to *Bacteroides, Clostridium coccoides*, and *Clostridium leptum* are the predominant bacterial groups (15). Given the complexity and multifactorial in terms of the evolution of the human intestinal microbiota, it is difficult to establish the composition of an ideal and healthy microbiota. Generally, a state of eubiosis is characterized by a strong presence of Firmicutes and Bacteroidetes and by a low percentage of Proteobacteria, which, instead, increase during inflammatory states (17). Another aspect that should be underlined is crosstalk between the gut microbiota and immune system. This point is extensive and critical. It allows for the tolerance of commensal bacteria and oral food antigens and also enables the immune system to recognize and attack opportunistic bacteria in order to prevent invasion and infection. In addition, microbiota has broader effects contributing to innate and adaptive immunity at multiple levels. This concept is supported in preclinical models, as germ-free mice lacking intestinal microbiota are subject to severe immunity defects, with a marked reduction of mucous layer, altered IgA secretion and reduced size and functionality of Peyer's patches and draining mesenteric lymph nodes (1).

## Fecal Microbiota Transplantation

**Figure 1:**
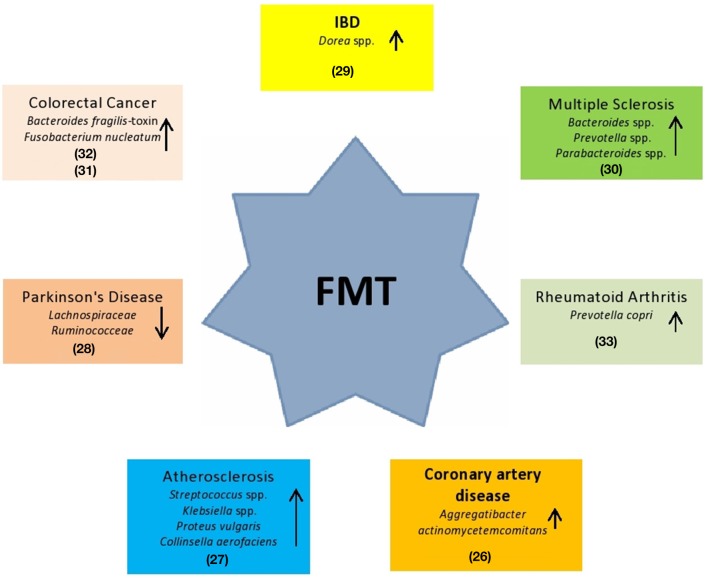
Potential use of FMT in systemic clinical disorders. These diseases are hypothesized being associated to specific gut bacteria alterations (26–33).

Given the fundamental role played by the human microbiota in the health/disease balance, the integrity of this system turns out to be an important therapeutic target (18). The most innovative therapeutic approach is represented by FMT. In the last decade, FMT has been an example of a valid solution, with success of ~90%, resulting in a more effective regimen for *Clostridium difficile* infection (CDI) than vancomycin (19). FMT consists of the infusion of a feces suspension from a healthy donor to the intestinal tract of a recipient patient in order to treat a specific disorder associated with alteration of gut microbiota (7, 20). In the European Consensus Conference (7), 28 experts from 10 countries collaborated to establish practice guidelines about FMT indications, donor selection, preparation of fecal suspension, clinical management, and basic requirements for implementing an FMT center. An aspect to highlight in FMT assessment is the healthy donor selection. First, potential donors have to undergo a medical interview to exclude risk factors. The main objective of donor selection is to reduce and prevent any adverse events related to the infused fecal material (21). Subsequently, serological and microbiological exams are performed on donor's fresh stool and blood. The aim is to avoid any possible infection. Laboratorists check for the presence of any pathogens, such as HIV, HBV, HCV, *Treponema pallidum*, Human T-lymphotropic virus I and II, *Plasmodium* spp., *Trypanosoma* spp., *Mycobacterium tuberculosis, Campylobacter* spp., *Escherichia coli* O157 H7, *Yersinia*, vancomycin-resistant enterococci (VRE), methicillin-resistant *Staphylococcus aureus* (MRSA), Gram-negative multidrug-resistant bacteria, *Norovirus*, antigens and/or acid fast staining for *Giardia lamblia* and *Cryptosporidium parvum*, protozoa (including *Blastocystis hominis*), helminths, and fecal occult blood testing. Regarding the laboratory preparation method, it is possible to choose between two different procedures: fresh and frozen preparations (22, 23). The first procedure requires that the feces of the donor be processed within 6 h after defecation to preserve the integrity of the anaerobic bacteria (24). Any amount of feces ranging from 30 to 50 g should be used and diluted in saline solution (NaCl 0.9%). The frozen approach consists of freezing fecal suspensions with the addition of glycerol at −80°C. Glycerol is necessary to preserve bacterial vitality during freezing (7). On the day of infusion, fecal suspension thawing should be performed at 37°C and then diluted to the desired volume by adding NaCl 0.9% (7, 25). Freezing feces is important for establishing a stool bank and the availability of stool on demand. Both methods have high efficacy (7). Promising findings suggest that FMT can also be used in the management of other extra-intestinal disorders associated with microbiota alterations. Some clinical conditions in which FMT could be a potential therapeutic method are described in Figure 1.

## Genital Female Microbiota Throughout Life

The vaginal district is a complex and essential ecosystem for female health and conception achievement/success. One aspect that needs to be observed is the microbial population residing in this region (34). In the last decade, metagenomic analysis has allowed researchers to outline the vaginal microbial ecosystem. The predominant species detected in the vagina of most healthy women are *Lactobacillus crispatus, Lactobacillus gasseri, Lactobacillus iners*, and *Lactobacillus jensenii* (35). Other important microbes found in healthy women are strictly anaerobic bacteria, such as *Atopobium, Gardnerella* (an opportunistic pathogen), *Megasphaera, Prevotella*, and *Peptoniphilus* (36). One important aspect is the dynamic shift that occurs across the female lifecycle and how it contributes to maintaining vaginal health. During perinatal development, the vaginal epithelium is thickened by residual maternal estrogen. This action allows for the deposition of glycogen in epithelial cells. Subsequently, glycogen is released by exfoliation of the epithelial cells, favoring glucose-fermenting microorganisms (37). Postnatally, when maternal estrogen is metabolized, the vaginal mucosa undergoes a thinning that leads to a reduction in glycogen and glucose-fermenting microorganisms and an ecological selection for a wide range of aerobes and facultative anaerobes (35). During childhood, microbiota is mostly populated by Gram-negative anaerobes, such as *Bacteroides, Fusobacterium, Veillonella*, and some Gram-positive anaerobes, including *Actinomyces, Bifidobacterium, Peptococcus, Peptostreptococcus*, and *Propionibacterium*. Regarding facultative anaerobic bacteria, *Staphylococcus aureus, Staphylococccus epidermidis, Streptococcus viridans*, and *Enterococcus faecalis* are mostly found (38). In prepubertal age, *Lactobacillus, Gardnerella vaginalis*, and *Prevotella bivia* are in low abundance. As described above, estrogen levels play a key role in vaginal bacteria colonization. Specifically, with the beginning of puberty, estrogen increases lead to further thickening, selecting for glucose-fermenting microorganisms. During adolescence, the microbiota evolves and becomes similar to the vaginal microbiota of adult women. In this stage, the dominant species are *Lactobacillus crispatus, Lactobacillus gasseri, Lactobacillus iners*, and *Lactobacillus jensenii* (38). The great decline of estrogen levels during menopause leads to a further switch in vaginal bacterial composition. In this stage of life, the microbiome is mainly characterized by *L. crispatus, L. iners, G. vaginalis*, and *Prevotella* and a lower abundance of *Mobiluncus, Staphylococcus, Bifidobacterium, Gemella*, and yeasts, such as *Candida* (39).

## Uterine and Cervicovaginal Microbiota and Local Factors

The uterus and vagina are in close anatomical proximity. For this reason, it is necessary to consider their microbial community as in continuous cross-talk. Cervicovaginal microbiota consists of 10^8^ bacteria/gram of vaginal fluid (40); it is decisive in women's health and reproductive outcomes. Despite being one of the simplest commensal bacterial communities in the human body, we are only beginning to appreciate its complex dynamic nature and important role in host immune modulation. Bacteria in the urogenital tract represent 9% of the total human microbiota, and most of them are difficult to culture. For almost a century, a common opinion stated that a healthy uterine cavity was germ-free (41). It was hypothesized that this sterility is guaranteed by the cervical plug, which constitutes an impermeable barrier to any bacteria coming from the vagina (42). In the last decade, many studies have challenged this assumption using culture-dependent methods and investigating the composition of the cervical mucous plug, which has been shown to not be impermeable to bacterial migration from the vaginal tract (35). In this review, our main question was whether to consider these microbes as “residents,” “tourists,” or “invaders.” The uterus and vagina are in strict continuity, allowing for an inevitable flow of bacteria to the uterine cavity and a dysregulation of uterine contractions, which may also promote bacterial colonization. Lactobacilli are fundamental for female reproductive tract homeostasis. The principal feature that makes lactobacilli defenders of the cervicovaginal niche is represented by their production of lactic acid and hydrogen peroxide, which maintain an acidic environment, with a pH ~4.0, which is inhospitable to the growth of catalase-negative bacteria. Ravel et al. (36), studied the cervicovaginal ecosystem among North American women of reproductive age using bacterial 16S rRNA gene sequencing. The results suggested the presence of five *community state types* (CSTs). CSTs I-IV are characterized by an abundance of *Lactobacillus* species (CST I, *L. crispatus*; CST II, *L. gasseri*; CST III, *L. iners*; CST V, *L. jensenii*), while CTS V consists mainly of anaerobes (36). *Lactobacillus*-dominant communities have shown important prevalence differences among healthy woman depending on their racial belonging. In fact, *Lactobacillus* spp. ranged from 90% in white women, 80% in Asian women and only 60% in Hispanic and black women. Moreover, it has been demonstrated that specific cervicovaginal bacteria are related to HIV acquisition. Specifically, in a South African study, a cohort of young women was screened in order to describe links between their vaginal microbiome and risk of acquiring HIV infection. The results showed that women with *L. crispatus*-dominant microbiota had a 4-fold lower risk of infection than women with highly diverse bacterial communities (43). Remarkably, not all *Lactobacillus* spp. are protective. For example, *Lactobacillus iners* increases pro-inflammatory cytokines, predisposing to infection. This action is implemented by a variety of other taxa, such as *Prevotella melaninogenica, Prevotella bivia, Veillonella, Mycoplasma*, and *Sneathia sanguinegens*, increasing the HIV acquisition rate (44, 45). A *Lactobacillus*-deficient cervicovaginal district is associated with higher genital pro-inflammatory cytokine levels and increased genital antigen-presenting cell activation via lipopolysaccharide (LPS)-sensing pathways and increased genital CD4^+^ T cell counts, acting as a “trigger” of local immunity (3, 43). Conversely, a vaginal ecosystem with a dominance of lactobacilli, particularly *L. crispatus*, is characterized by a low inflammatory microenvironment. Endocervix and a thick mucous layer represent very important elements that contribute to innate immune barriers (46). In the mucous, we can find the presence of IgG, secretory IgA, lactoferrin, lysozyme, and other antibacterial compounds. Moreover, T-cells and antigen-presenting cells are also in the lower reproductive endothelium.

Several studies have reported conflicting findings on the beneficial activity by *Lactobacillus*abundance. Higher levels were found in groups of women with endometrial polyps (EP) or in women with EP associated with chronic endometriosis (EP + CE) compared with healthy controls (47).

In contrast, other studies suggested that high levels of *Lactobacillus* are significantly associated with increased reproductive success in women undergoing *in vitro* fertilization (IVF) (48).

In recent years, many studies have shown how bacterial communities can play a key role in fecundation (42, 49). These new considerations are decisive for the development of new approaches to improve fertility, conception, healthy pregnancy, and bacterial colonization of the infant and to prevent preterm birth. Considering this strong relationship between microbes and fertility, the main objective must be to intervene directly on these populations in order to increase reproductive success outcomes. An example of this strategy is represented by the application of the probiotics *Lactobacillus brevis CD2, Lactobacillus plantarum FV9*, and *Lactobacillus salivarius FV2*. These species seem to exercise a protective action on sperm motility and viability *in vitro* by preventing membrane damage induced by reactive oxygen species (35). This suggests that lactobacilli species resident in the vagina could be a protective factor for spermatozoa function during and after intercourse. This relationship has also been observed in artificial reproduction technique (ART) outcomes. In fact, vaginal microbiota composition, on the day of embryo transfer and after IVF, influenced the success of pregnancy (50). As described before, some disorders strongly alter cervicovaginal flora. Uterine microbiota composition has been shown to be significantly different in women with endometriosis (51). Furthermore, Cicinelli et al. reported that endometriosis patients treated with antibiotics before implantation had significantly better reproductive outcomes compared with those not treated (52). Endometriosis patients are characterized by low levels of *Lactobacillus* spp. and an increase of *Streptococcus* spp., *Staphylococcus* spp., and Gram-negative bacteria belonging to the family *Enterobacteriaceae*. This relationship between bacteria, endometriosis and reproductive outcomes has been studied, demonstrating how a “*Lactobacillus* dominant (LD)” population and a “non-*Lactobacillus* dominant” (NLD) show significant differences in terms of successful outcomes. Specifically, women with an LD uterine microbiome had markedly higher rates of implantation [60.7 vs. 23.1% (*P* = 0.02)], pregnancy [70.6 vs. 33.3% (*P* = 0.03)], ongoing pregnancy [58.8 vs. 13.3% (*P* = 0.02)], and live births [58.8 vs. 6.7% (*P* = 0.002)] compared with those with an NLD uterine microbiome composition (53). Furthermore, researchers are studying the functional impact of uterine microbial population and their relationship with the microenvironment. The promotion of vaginal health is influenced by probiotics with various combinations of *L. reuteri L. acidophilus, L. gasseri, L. rhamnosus, L. plantarum*, and *L. crispatus*. When given as a short course (3–10 days), after 1 month, rates of vaginal colonization by probiotic strains ranged from 10% for oral *L. rhamnosus* to 53% for a vaginal product, including *L. gasseri, L. fermentum, L. rhamnosus*, and *Pediococcus acidilactici*. Treatment with a vaginal *L. crispatus* product established persistent colonization up to 1 month in 44%−59% of women (46). However, 1 week after the treatment, the rates of colonization decreased in sexually active women and in women yet colonized by endogenous lactobacilli (54). Another potential approach to modulate cervicovaginal microbiota is hormone therapy. In 2013, van de Wijert et al. (55) observed that combined oral contraceptives and progestin-only injectable contraceptives were mainly associated with a lower incidence of bacterial vaginosis (BV). In post-menopausal women, when the concentration of *Lactobacilli* was reduced, it was observed that treatment with oral estradiol hormone replacement therapy seemed to influence vaginal microbiota, increasing colonization by *Lactobacillus* spp. (56).

## Prospective FMT Treatment of Female Genital Tract Diseases

The role of commensal bacteria in stimulating local and systemic immunity appears to be established. Some of the new strategies for the treatment of diseases are focused on chemically synthetized molecules or recombinant proteins (57), and this type of approach requires oral or topical administration. Moreover, such compounds frequently show low levels of stability and, sometimes, in cases of recombinant proteins, contain remnants of inflammatory contaminants (58). Bacterial strains resident in the gut and vagina crosstalk, which leads to local and systemic immune regulation. In this regard, particular bacterial species can be engineered and used as oral vectors capable of triggering local and systemic immune responses in order to prevent (e.g., vaccines) or alleviate disease symptoms (59).

Induction of humoral and cellular immune responses represents a fundamental aspect involved in preventing female genital tract disorders. As other secretions, vaginal and cervical fluid contain mainly IgA and a certain proportion of IgG of plasma origin (60). The female genital tract does not contain organized lymphoepithelial structures analogous to intestinal Peyer's patches. This aspect is crucial to identify alternative routes of immunization to bypass local application of antigens. Thus, oral, rectal and nasal administrations were suggested (61). Furthermore, a central aspect is represented by the homing of activated lymphocytes, which depends on the expression of specific surface receptors that recognize elements on endothelial cell walls. An example is α*4*β*7* and *L-selectin* receptor, which guide immune cells to the gut mucosa and peripheral lymph nodes, respectively (62). An intriguing study was conducted in 2001 by Kutteh et al. They explored the efficacy of intestinal tract immunization in the induction of specific antibodies in human female genital tract secretions. A live attenuated strain of *Salmonella typhi* Ty 21a was used as a vector. In this study, 15 women were vaccinated orally, and 11 volunteers received the same dose rectally. To evaluate the effect on mucosal responses, seven women that had been vaccinated orally 6 months prior received a rectal boost. The data showed significant increases of IgA, predominantly, and IgG in vaginal and cervical fluids. Increased immunoglobulin levels were also observed after rectal boosting. This demonstrated that specific antibodies in the female genital tract induced by primary vaccination could be enhanced via subsequent rectal administration (60). Two aspects clearly emerged from this study: (1) the administration of live bacterial vectors induces a local and systemic immune response; (2) rectal administration may also induce stimulation at the cervicovaginal level. In this regard, FMT could be a natural alternative to recombinant bacterial vectors. In fact, with single or multiple infusions, large amounts of bacteria and metabolites could be administered, each of which could serve as an inducer of local and systemic immune responses.

Indeed, understanding the connection between intestinal and vaginal microbiota may represent a goal for new treatments of female genital tract disorders. New gene sequencing techniques have allowed characterization of the intestinal and vaginal populations and therefore led to hypotheses of interactions in states of health or disease. In 2017, Lindheim et al. investigated stool microbiome of women with polycystic ovary syndrome (PCOS) and healthy controls. PCOS is a common disorder affecting 5–10% of women in their reproductive years. As far as we know, this syndrome is characterized by the presence of three main features: hyperandrogenism, oligo/anovulation, and polycystic ovaries on pelvic ultrasound (63). The etiology and pathogenesis of PCOS remain unclear and may be multi-factorial, involving genetic, neuroendocrine, and metabolic causes (64). Other parameters, such as gut barrier integrity, endotoxaemia and inflammation, were also evaluated. The stool microbiome of PCOS patients showed a lower diversity and an altered phylogenetic composition compared to controls. Significant differences in taxa, with a relative abundance >1%, were not observed (65). Regarding rare taxa, the relative abundance of bacteria from the phylum Tenericutes (relevant genera include *Mycoplasma* spp., *Ureaplasma* spp.) and the family S24-7 (phylum Bacteroidetes) was significantly lower. Moreover, patients did not show alterations in all markers of gut barrier function and endotoxaemia (65). These findings suggest that changes of gut microbiota also had trends similar to the variations of metabolic symptoms. Other studies observed that some Gram-negative bacteria belonging to the genera *Bacteroides* and *Escherichia/Shigella* significantly increased in the gut of PCOS women with obesity. In these conditions, LPS produced by these microorganisms was demonstrated to induce chronic inflammation, obesity, and insulin resistance in LPS-infused mice (5). In this status, spore-forming species, such as *Clostridiales*, decrease, while Proteobacteria and Bacteroidetes increase. Other evidence suggests a multifactorial relationship in determining the shape of cervicovaginal microbiota. Recently, a potential relationship between sex hormones and gut microbiota emerged. This novel concept has been defined as “*microgenderome*” (66). At the time of puberty, sex hormone levels exercise specific influences on the composition of the microbiota. Removal of gut microbiota showed an increase in testosterone concentration in female mice but a decrease in male mice. Thus, the commensal gut microbiota also had effects on production of the male sex hormone (67). It is interesting to explore the role of gut microbiota in PCOS, in which androgen levels in PCOS women are always elevated. Tremellen and Pearce suggest that dysbiosis of the gut microbiota (DOGMA) brought about by a high fat-sugar diet in PCOS patients leads to an increase in intestinal permeability. Lipopolysaccharide produced by Gram-negative bacteria traverse the gut wall to enter the circulation, leading to a chronic state of low-grade inflammation. Activation of the immune system interferes with insulin receptor, driving up insulin levels, which boost testosterone production in the ovary, leading to PCOS. DOGMA theory can account for the role of gut microbiota in the pathogenesis of PCOS (68). This evidence has been reinforced by a study on induced PCOS using a rat model (67), in which the results showed that PCOS rats displayed abnormal oestrous cycles, represented by increasing androgen biosynthesis, and exhibited multiple large cysts with diminished granulosa layers in ovarian tissues. Moreover, the composition of gut microbiota in rats treated with letrozole, a non-steroidal aromatase inhibitor, was different from controls. *Lactobacillus, Ruminococcus*, and *Clostridium* species were lower, while *Prevotella* species was higher in PCOS rats compared with control rats. The rats were treated using FMT from healthy rats. It was found that the oestrous cycles were improved in all rats in the FMT group, with decreased androgen biosynthesis and normalized ovarian morphologies. The composition of the restored gut microbiota in the FMT group was characterized by an increase in *Lactobacillus* and *Clostridium* and a decrease in *Prevotella*. This evidence indicated that the dysbiosis of gut microbiota was associated with the pathogenesis of PCOS and emphasized the hypothesis aimed at implementing the use of FMT for extra-intestinal disorders. In fact, there is strong evidence that host-microbial interactions play a key role in reproductive health outcomes. The challenge is to find tools and strategies capable of modulating microbial populations in the cervicovaginal microenvironment, using probiotics, as well (46). Other female tract disorders are associated with gut microbiota alterations, such as endometriosis and BV.

Endometriosis is a condition defined by the presence of endometriotic gland and stroma outside of the uterine cavity, ranging in severity levels from I to IV (69). Many studies have emphasized how immunologic alterations and microbiological factors, independently or in association with epigenetic changes, might play a role in the development of the disease (70). Dysbiosis influences estrogen levels in the circulation (71), and increased estrogen levels can stimulate growth of ectopic endometriotic foci and inflammatory activity. Thus, gut microbiota influencing the regulation of the estrogen cycle could be associated with endometriosis. In an interesting recent study, Ata et al. performed an in depth characterization and comparison of vaginal, cervical, and gut microbiota in women with stage III–IV endometriosis and healthy controls. The results showed overall comparable compositions of the vaginal, cervical, and gut microbiota. However, there were some differences between the bacterial groups, with some interesting decreases, absences or increases of some species. Specifically, the complete absence of *Atopobium* in the vagina and cervix, together with the increased abundance of *Gardnerella, Escherichia/Shigella*, and *Ureoplasma* in the cervical microbiome of patients with endometriosis could be a relevant finding (72). *Atopobium* spp. was recently implicated as a gynecological pathogen associated with bacterial vaginosis, obstetric bacteraemia, and, possibly, with endometrial cancer (73). These findings suggest an active role played by resident bacteria in the cervix-vaginal district and interactions with intestinal microbes. This bacterial network appears to be related to local and system female conditions.

**Figure 2:**
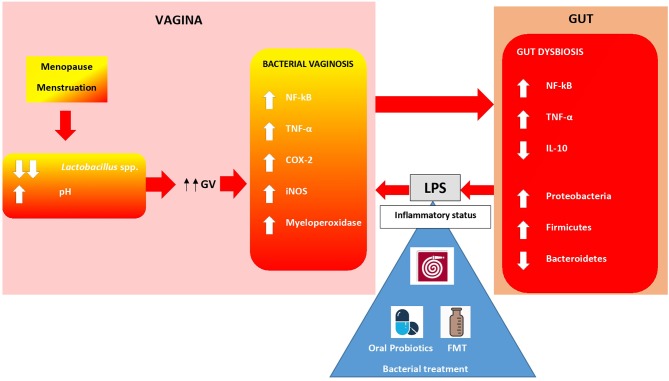
Gut-Vagina cross-talking. Bacteria and immunity cells in vagina and gut are in close communication. Hormonal changes during menopause, menstruations lead to an overgrowth of anaerobic species such as *Gardnerella vaginalis* (GV). Bacterial vaginosis (BV), caused by GV, increases an immunity molecules release which exerts pro-inflammatory effects *in situ* and in the gut. Proteobacteria overgrowth leads to LPS iper-production establishing an inflammatory status. Oral probiotics alleviate BV in women. FMT might play a similar role in regulating both altered gut flora and vagina inflammation (74, 77).

As previously mentioned, menstruation and menopause, caused by hormonal changes, contribute to a drastic modification in the vaginal microbiota, specifically decreases in lactobacilli. In this condition, infections by *Gardnerella vaginalis* (GV) and *Candida albicans* are promoted (74). GV plays a key role in vaginal immunity. In fact, this bacteria is able to increase levels of several inflammatory factors, such as NF-kB, TNF-α, myeloperoxidase activity, COX-2, and iNOS. This pattern of molecules leads to BV (75, 76). Some studies have shown how the oral administration of probiotics can influence immunity in the vagina (77). Probiotics are a valid alternative to antibiotic treatment in order to avoid antimicrobial resistance. Data suggest that strains of *L. acidophilus, L. rhamnosus* and *L. johnsonii* mitigate GV-vaginosis in mice (78). Kim et al. have demonstrated that administration of NK3 *(L. plantarum*), NK49 (*B. longum*), and their mixture alleviated GV-induced BV in mice by reducing TNF-α levels and myeloperoxidase activity and increasing IL-10 expression. This treatment also contributed to COX-2, iNOS and NF-Kb suppression (77). In this study, the authors also investigated fecal microbiota composition in mice with GV-induced BV using qPCR. High levels of GV significantly increased Firmicutes and Proteobacteria, the main LPS-producers, and suppressed Bacteroidetes populations in the vagina. Moreover, GV infection was able to increase inflammatory molecules, such as TNF-α, NF-Kb, and myeloperoxidase, in the colon. However, treatment with NK3 and/or NK49 significantly decreased Proteobacteria and increased Bacteroidetes, contributing to the inhibition of gut microbiota LPS production (Figure 2). Crosstalk between the gut and vagina environment seems evident. Oral administration of probiotics, and then bacteria, might play a crucial role in the suppression of the pro-inflammatory cytokine expression in the vagina. This action influences the inflammatory status in the gut, acting on bacteria and immune cells, such as macrophages, dendritic cells and local lymphocytes. It could be interesting to hypothesize a similar action using FMT, which would allow a direct administration of bacteria and metabolites in the gut, bypassing stomach passage.

## Concluding Remarks

Undoubtedly, the countless microbial communities present on the body's surface and in internal organs are directly involved in homeostasis (2). Knowledge into the interactions between the intestinal microbiota and health/disease balance, due to immune-related actions that influence extra-intestinal sites and diseases, is deepening. Concerning vaginal districts, evidence indicates that the immune system is involved in local disorders (59). Indeed, this relationship could be compellingly linked to gut microbes. On the other hand, vaginal microbiota represents a complex biological niche, contributing to the local health and coinciding with the anatomical intricacy of the female genital tract. Its proximity and connection to the intestine allows for strong interactions between gut and vaginal bacteria. These aspects must also consider the numerous chemical changes that the female reproductive tract undergoes with age and, periodically, with hormonal interactions (35). Then, a refined equilibrium between gut microbiota, immunity, vaginal microbiota, and hormones is essential in the physiological state of the female genital tract. Considering recent literature evidences, a direct action exerted by oral probiotics in the modulation of vaginal disorders such as BV appears clear (78). Another important finding was the association of specific patterns of intestinal bacteria with vaginal pathologies such as endometriosis and PCOS (67, 72). Alongside the synergistic action of intestinal and vaginal bacteria, the hormones, such as oestrogens, influence the cervicovaginal microenvironment (66). It is necessary to continue focusing on the dynamics of these interactions and on the effects on health status or diseases. The new tools developed in modern microbiology, such as metagenomic and culturomic analysis, must lead to a greater understanding of microorganism-host interactions and modify the simple concept of “commensal” or “pathogen” (8). The right way is to enhance the biological context in which the bacterial species live and the biological pathways they control. Gut bacteria are an “orchestra” exerting mechanisms of regulation on extra-intestinal bacteria, circulating hormone levels, and immunity. Indeed, manipulating the microbiome composition is a potential strategy to prevent or mitigate several diseases. The next step for the scientific community must be to clarify what is “normal” in the human microbiome, including cervicovaginal microbes, in order to define dysbiotic, disease-associated profiles, with the aim of providing therapies, or lifestyle interventions to return it to normal again.

A concrete future prospect could involve “driving” the bacterial populations that inhabit these organs through therapeutic procedures, such as fecal transplantation, as yet demonstrated in CDI resolution (7). FMT consists of a liquid feces suspension, infused into a recipient colon-site. In this way, bacteria and metabolites are able to modulate both local microbial community and systemic immunity, influencing other districts balances, as hopefully the female genital-tract one. An advanced, intriguing possibility could involve the infusion of a synthetic bacterial suspension consisting exclusively of selected and controlled bacterial populations in order to obtain an “*ad personam*” therapeutic approach.

## Author Contributions

GQ, MS, and LM drafted the manuscript and conducted the literature review.

### Conflict of Interest

The authors declare that the research was conducted in the absence of any commercial or financial relationships that could be construed as a potential conflict of interest.
